# Draft Genome Sequence of Highly Nematicidal *Bacillus thuringiensis* DB27

**DOI:** 10.1128/genomeA.00101-14

**Published:** 2014-02-20

**Authors:** Igor Iatsenko, Craig Corton, Derek J. Pickard, Gordon Dougan, Ralf J. Sommer

**Affiliations:** a Max Planck Institute for Developmental Biology, Department of Evolutionary Biology, Tübingen, Germany; b Wellcome Trust Sanger Institute, Wellcome Trust Genome Campus, Hinxton, Cambridge, United Kingdom

## Abstract

Here, we report the genome sequence of nematicidal *Bacillus thuringiensis* DB27, which provides first insights into the genetic determinants of its pathogenicity to nematodes. The genome consists of a 5.7-Mb chromosome and seven plasmids, three of which contain genes encoding nematicidal proteins.

## GENOME ANNOUNCEMENT

*Bacillus thuringiensis* is a Gram-positive bacterium pathogenic to a number of invertebrate hosts (1). The pathogenicity of *B. thuringiensis* is largely mediated by crystal (Cry) toxin proteins produced during sporulation (2). Due to the insecticidal properties of the Cry toxins, which are not toxic to vertebrates, *B. thuringiensis* has been extensively used as a biopesticide (1, 2).

Whole-genome sequencing has proven to be a useful tool for the discovery of novel Cry toxins (3). While several *B. thuringiensis* strains have been sequenced (4–9), there is lack of whole-genome sequence data for nematicidal *B. thuringiensis* strains. Obviously, such information is of great importance, since it helps to reveal unknown aspects of *B. thuringiensis* pathogenic mechanisms, e.g., pathogenicity against animals other than insects. One potential target is nematodes, which often live in the soil and were previously shown to interact with *Bacillus* Cry toxins (10).

We previously isolated the nematicidal strain *B. thuringiensis* DB27 from dung beetles, and this strain exhibits strong virulence to the nematode *Caenorhabditis elegans* (11). While the mechanisms of *C. elegans* resistance (12) and transcriptional response (13) to this pathogen have been described, the nematicidal virulence determinants of *B. thuringiensis* DB27 are currently unknown. To provide first insights into *B. thuringiensis* DB27 virulence mechanisms, we sequenced its whole genome.

Genomic DNA was isolated from *B. thuringiensis* DB27 using the MasterPure Gram-positive DNA isolation kit (Epicenter). Whole-genome sequencing was performed using Roche and Illumina platforms with a GS FLX Titanium 8-kb paired-end library and an Illumina 250-bp paired-end library, respectively. Approximately 2.4 million 150-bp Illumina reads were assembled using Velvet version 1.1.06 (14). The resulting Velvet assembly was combined with ~280,000 454 reads, with an average length of 308 bases, using Newbler *de novo* version 2.6 assembler, generating a total of 387 contigs, 260 of which were contained in 33 scaffolds representing 98.4% of the total genome assembly. The combined assembly was then improved using computational and manual methods: (i) IMAGE (15) was used for the Newbler-generated scaffold information and Illumina reads were used to reduce the number of sequence gaps, (ii) ICORN (16) used Illumina data to correct base errors introduced by 454 sequencing, and (iii) the sequence was manually edited in Gap4 (17). The final assembly is represented by 235 contigs, of which 156 contigs are in 7 scaffolds representing the main chromosome, 49 contigs are unplaced, and 30 contigs are in 7 scaffolds identified as plasmids. The open reading frames (ORFs) were identified using Prodigal version 2.6 (18).

The genome of *B. thuringiensis* DB27 consists of a 5.7-Mb chromosome and seven plasmids ranging in size from 4 to 200 kb. The G+C content of the chromosome is 35.2%, and that of the plasmids ranges from 31.5% to 34.4%. The total number of predicted genes is 6,302, with 5,851 genes located on the chromosome and 451 genes on the plasmids. Toxin genes were identified using BtToxinScanner (3). In total, 3 Cry-like genes belonging to the Cry21 nematicidal family were identified as being carried by 200-kb, 8-kb, and 6-kb plasmids.

### Nucleotide sequence accession numbers.

The draft of the whole-genome sequencing project has been included in the European Nucleotide Archive at EMBL-EBI under accession no. CBXL010000001 to CBXL010000235.
